# Infantile Subacute Brainstem Infarction and the Presence of Incidental Ectopic Lymphoid Tissue: A Rare Case

**DOI:** 10.7759/cureus.95753

**Published:** 2025-10-30

**Authors:** Ambrose Loc T Ngo, Miguel Guzman, Deiter Duff

**Affiliations:** 1 Osteopathic Medicine, Kansas City University, Joplin, USA; 2 Pathology, Saint Louis University School of Medicine, St. Louis, USA; 3 Forensic Pathology, Greene County Medical Examiner's Office, Springfield, USA

**Keywords:** brain infarct, neonatal neurology, neuro-critical care, stroke, young-onset stroke

## Abstract

Unexpected infant deaths often present complex diagnostic challenges, where clinical histories alone may not reveal the underlying etiology. Autopsy examination remains essential in identifying uncommon neuropathologic findings that contribute to mortality.

A two-month-old male infant was found unresponsive in his car seat (day 0) and succumbed four days later despite intensive medical intervention. His medical history included neonatal respiratory distress with two prior admissions to the neonatal intensive care unit. Autopsy was conducted one day after death (approximately five days postictus), revealing ectopic lymphoid tissue within the medulla of the brainstem, accompanied by subacute infarction and early necrosis. Additional systemic findings included acute splenic infarction, colonic inflammation, and focal acute bronchiolitis. Genetic analysis (collected two days postictus) identified a heterozygous *SLC4A3* missense variant, though it was not deemed contributory to death.

This case describes a rare neuropathologic finding of ectopic lymphoid tissue within the brainstem of an infant, suggesting an underrecognized cause of death. It underscores the indispensable role of autopsy in elucidating rare pediatric neuropathologies and broadening the understanding of infant mortality mechanisms.

## Introduction

Cerebrovascular accidents, or strokes, affect over 795,000 individuals annually in the United States [1]. Although stroke can occur at any age, the mean age of onset is 74.3 years, with approximately 62% of hospitalized cases occurring in patients over 65 years of age [2,3]. In contrast, stroke in the pediatric population remains exceedingly rare, with an estimated incidence of 1.3 per 100,000 children and 9.6 per 100,000 infants compared to 269 per 100,000 adults [4-7]. Most strokes occur within the middle cerebral artery distribution and its branches, while infarctions involving the brainstem account for only about 10% of both ischemic and hemorrhagic strokes across age groups [8-10]. These epidemiologic findings underscore the exceptional rarity of infantile brainstem infarction, a condition associated with particularly high morbidity and mortality given the brainstem’s critical role in autonomic, respiratory, and motor regulation. Ectopic lymphoid tissue (ELT) refers to organized lymphoid aggregates that arise outside primary lymphoid organs, such as the spleen or lymph nodes, often in response to chronic inflammation, infection, autoimmune processes, or neoplasia [11-13]. While ELT has been described in organs such as the lungs, salivary glands, and joints, reports of ELT within the central nervous system (CNS) are scarce, particularly in neonates and infants. In adults, ELT has been associated with multiple sclerosis and primary CNS neoplasms, but its significance in pediatric neuropathology remains poorly understood. Importantly, ELT can occasionally occur without an identifiable cause, and its clinical implications may vary depending on context and location.

Here, we present a rare case of infantile brainstem infarction with concurrent ELT within the medulla, identified at autopsy following the sudden death of a two-month-old infant. To our knowledge, no prior cases describing the coexistence of brainstem infarction and ELT in infants have been documented, highlighting the value of postmortem examination in uncovering unusual neuropathologic findings and expanding understanding of unexplained infant mortality.

## Case presentation

A two-month-old male infant was found unresponsive and pulseless in his car seat (day 0). Resuscitation efforts were successful, and he was transported to the hospital, where intensive care support, including intubation, was required. Despite aggressive medical management, the patient was pronounced dead on hospital day 4 (four days postictus).

Perinatal and medical history

The infant had a history of neonatal respiratory distress requiring two prior admissions to the neonatal intensive care unit. He was born vigorous but developed apnea approximately five minutes after delivery, with oxygen desaturations into the low 80s. Two weeks before his death, the patient experienced an episode consistent with croup.

Screening and laboratory findings

Initial metabolic screening (collected on day 2 postictus) revealed elevated acylcarnitines, amino acids, and carnitine levels. These findings suggested the possibility of a primary or secondary carnitine metabolism abnormality; however, no overt inherited metabolic disorder was identified. It was noted that these values can also be transiently altered during acute illness, limiting diagnostic specificity.

Imaging and hospital course

Computed tomography of the chest, abdomen, pelvis, cervical spine, and head (obtained day 0 postictus) demonstrated no acute abnormalities. Echocardiography revealed normal cardiac structure and function.

Autopsy and neuropathologic findings

**Figure 1:**
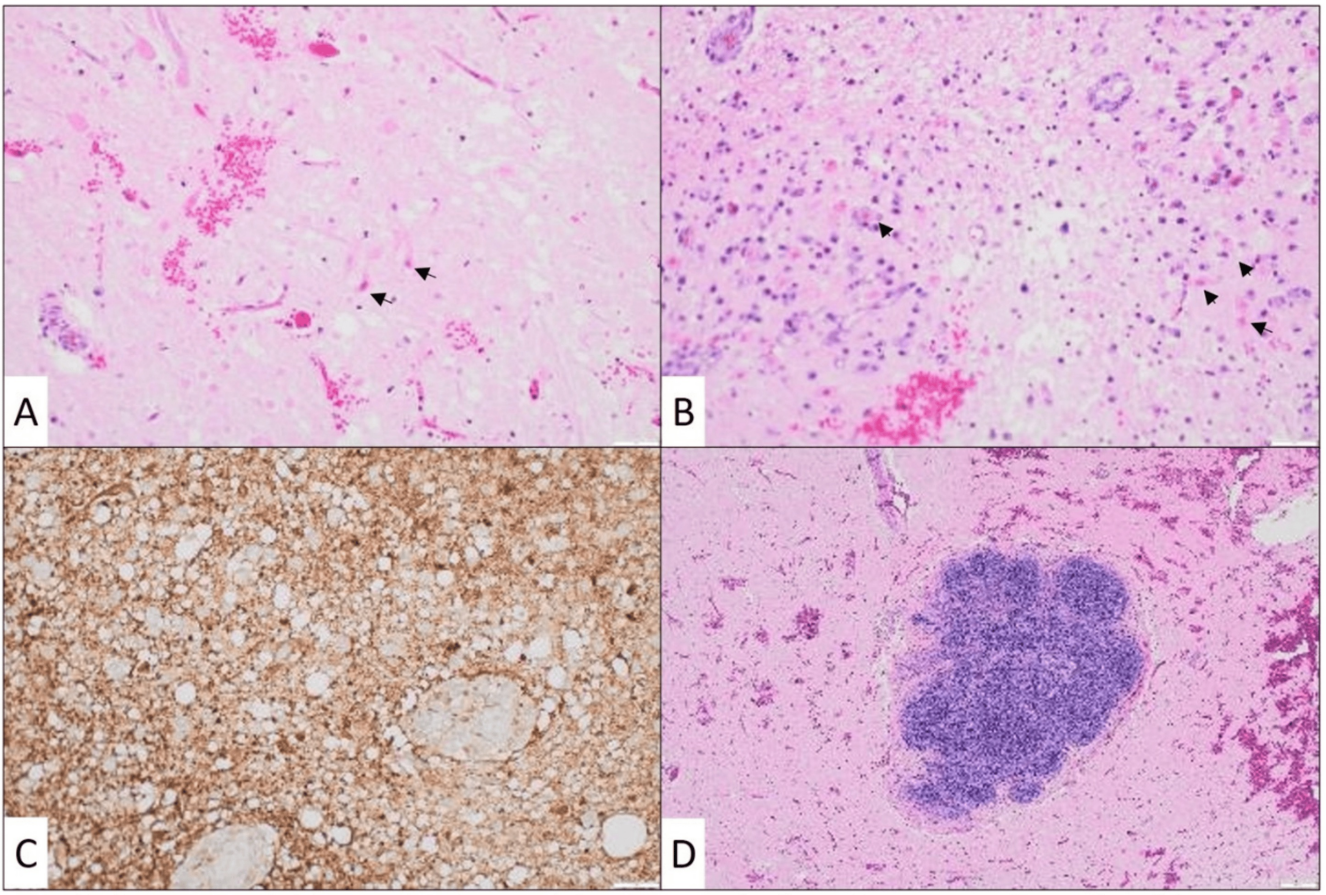
Neuropathological findings Microscopic examination of the brainstem, specifically the medulla, revealed ischemic/hypoxic neurons (black arrows) in areas of neuropil rarefaction, neuronal loss, numerous macrophages, and microglial activation (A, B: H&E, original magnification 20X). Immunohistochemical stain for glial fibrillary acidic protein (GFAP) demonstrated extensive gliosis in the areas of subacute infarction (C: GFAP, original magnification 20X). An incidental, intraparenchymal lymphoid aggregate was also identified (D: H&E, original magnification 4X).

Postmortem examination was conducted one day after death (approximately five days postictus), which revealed a normally developed male infant with mild diastasis of the cranial sutures secondary to cerebral edema. The medulla of the brainstem showed brown discoloration, and histologic sections were referred for neuropathologic consultation. Microscopic evaluation of the medulla revealed features consistent with subacute infarction, including neuronal loss, neuropil rarefaction, numerous macrophages, microglial activation, and early gliosis (Figure 1). No glial or hematopoietic neoplasm was identified. Additionally, ELT was observed incidentally within the medulla. There was no histologic evidence of vasculitis or vascular occlusion to suggest a specific cause for the infarction. Other systemic findings were consistent with post-cardiac arrest changes and complications of hospitalization, including ischemic changes in the colon and spleen, foci of diffuse alveolar damage, and very focal acute bronchiolitis.

Genetic findings

Genetic analysis revealed a heterozygous SLC4A3 gene missense variant (c.2716A>T). The SLC4A3 gene encodes an anion exchanger (AE3) involved in chloride-bicarbonate transport, critical for maintaining intracellular pH and cardiac electrophysiologic stability. While mutations in this gene have been associated with short QT syndrome, the identified variant was determined to be of uncertain significance and not contributory to death, as the brainstem infarction was the clear proximate cause.

## Discussion

This case describes a subacute brainstem infarction as the cause of death in an infant found unresponsive in a car seat. Brainstem infarctions in infants are rare and often under-recognized, with clinical manifestations that may overlap with hypoxic-ischemic injury [14,15]. A definitive etiology for the infarction, such as a vascular occlusion, was not identified. Moreover, the distribution of injury did not correspond to a single vascular territory but was instead consistent with a tegmental watershed-type infarction. The differential diagnosis primarily included hypoxic-ischemic injury secondary to respiratory compromise versus primary vascular occlusion. However, the absence of histologic evidence of thromboembolism, vasculitis, or vascular malformation supports a hypoxic-ischemic mechanism as the most plausible explanation. The infant’s clinical history of early respiratory difficulties, leading to two neonatal intensive care unit admissions, suggests possible intermittent hypoxia or impaired perfusion, predisposing to progressive ischemic vulnerability of the medulla.

It was also reported that the patient suffered from croup two weeks before death, raising the possibility of a preceding post-infectious or immune-mediated process. In this context, the presence of ELT within the medulla represents an unusual but potentially meaningful finding. Ectopic lymphoid aggregates within the CNS have been described in chronic inflammatory and autoimmune diseases, such as multiple sclerosis and CNS neoplasms, though their occurrence in infants and within the brainstem is exceedingly rare [16,17]. The ELT observed in this case may reflect a localized immune response or reactive lymphoid neogenesis following infection or hypoxic stress, rather than a direct causal factor in the infarction itself. Nonetheless, such findings broaden the understanding of how immune activation might accompany or follow ischemic injury in the developing brain.

Genetic testing identified a heterozygous missense variant in the SLC4A3 gene (c.2716A>T), a variant of uncertain significance. The SLC4A3 gene encodes an anion exchanger (AE3) involved in chloride-bicarbonate transport, contributing to intracellular pH regulation and cardiac electrophysiologic stability. Although variants in SLC4A3 have been implicated in short QT syndrome and other cardiac arrhythmias, the clinical relevance of such variants in pediatric populations remains poorly defined [18]. In this case, the genetic finding was considered incidental and non-contributory to death, emphasizing the importance of correlating molecular data with autopsy findings to prevent overinterpretation of variants of uncertain significance.

From a diagnostic and clinical standpoint, this case highlights the continued importance of comprehensive autopsy and neuropathologic evaluation in unexplained infant deaths. Recognition of atypical findings such as ELT within the brainstem may prompt further postmortem immunologic and genetic investigations, aiding in the identification of subtle or novel mechanisms underlying pediatric cerebrovascular pathology. Collaboration between clinicians, pathologists, and geneticists is essential to contextualize such findings and prevent premature attribution of causality to genetic variants or incidental histologic changes.

Limitations

A key limitation of this report is the inability to establish a definitive etiologic pathway for the brainstem infarction due to the absence of specific vascular injury or isolated infectious organisms. Although ELT was clearly present, its clinical significance remains uncertain without supporting functional or immunologic studies. Additionally, no inflammatory marker assays, viral polymerase chain reaction (PCR) testing, or molecular autopsy analyses were performed, which limits the ability to evaluate subtle immune-mediated or infectious contributions to the observed pathology. Targeted viral PCR or immunohistochemical staining may have provided greater clarity regarding the potential role of infection or post-infectious inflammation in lesion development. Genetic testing was also limited to a panel-based approach, potentially overlooking non-coding or novel variants of relevance. Furthermore, while clinical observations suggest possible interactions between early respiratory compromise, infection, and cerebrovascular vulnerability, these hypotheses remain speculative given the retrospective and single-case nature of this report.

Future directions

Further research should focus on understanding the role of ELT within the developing CNS and determining whether such findings are more prevalent among infants with unexplained brainstem pathology than previously recognized. Additionally, investigating the subtle interplay between minor respiratory infections, genetic predispositions, and cerebrovascular fragility in neonates may uncover mechanisms underlying rare pediatric strokes. Given the complexity of these cases, a multidisciplinary approach involving neuropathology, genetics, and pediatrics is essential to achieve comprehensive evaluation and interpretation of such findings. Due to the infrequency and multifactorial nature of these presentations, there is also a continued need for centralized pediatric neuropathology registries and broader multi-institutional collaborations to enhance data collection, promote diagnostic consistency, and advance understanding of these rare but significant pathologies.

## Conclusions

Overall, this case illustrates the need for thorough investigations after death, particularly when imaging and clinical workup are inconclusive in pediatric mortality. It highlights the importance of neuropathological consultation and autopsy in ascertaining the cause of death, developing future clinical and genetic interventions, and preventing misattributions.

## References

[1] Tsao CW, Aday AW, Almarzooq ZI (2023). Heart disease and stroke statistics-2023 update: a report from the American Heart Association. Circulation.

[2] Jackson G, Chari K (2019). National hospital care survey demonstration projects: stroke inpatient hospitalizations. Natl Health Stat Report.

[3] Akyea RK, Vinogradova Y, Qureshi N (2021). Sex, age, and socioeconomic differences in nonfatal stroke incidence and subsequent major adverse outcomes. Stroke.

[4] Lee KL, Tseng YC, Yang ST, Kuo YT (2018). Uncommon pediatric stroke caused by MCA dissection presenting as initial loss of consciousness. Pediatr Neonatol.

[5] Earley CJ, Kittner SJ, Feeser BR (1998). Stroke in children and sickle-cell disease: Baltimore-Washington Cooperative Young Stroke Study. Neurology.

[6] Williams GR (2001). Incidence and characteristics of total stroke in the United States. BMC Neurol.

[7] Nichols L, Bridgewater JC, Wagner NB, Karivelil M, Koelmeyer H, Goings D, Hope TD (2021). Where in the brain do strokes occur? A pilot study and call for data. Clin Med Res.

[8] Gowda SN, Munakomi S, De Jesus O (2025). Brainstem stroke. StatPearls [Internet].

[9] Carragher DM, Rangel-Moreno J, Randall TD (2008). Ectopic lymphoid tissues and local immunity. Semin Immunol.

[10] Bogousslavsky J, Van Melle G, Regli F (1988). The Lausanne Stroke Registry: analysis of 1,000 consecutive patients with first stroke. Stroke.

[11] van de Walle T, Vaccaro A, Ramachandran M, Pietilä I, Essand M, Dimberg A (2021). Tertiary lymphoid structures in the central nervous system: implications for glioblastoma. Front Immunol.

[12] Aloisi F, Pujol-Borrell R (2006). Lymphoid neogenesis in chronic inflammatory diseases. Nat Rev Immunol.

[13] Manzo A, Bombardieri M, Humby F, Pitzalis C (2010). Secondary and ectopic lymphoid tissue responses in rheumatoid arthritis: from inflammation to autoimmunity and tissue damage/remodeling. Immunol Rev.

[14] Sugama S, Eto Y (2003). Brainstem lesions in children with perinatal brain injury. Pediatr Neurol.

[15] Quattrocchi CC, Fariello G, Longo D (2016). Brainstem tegmental lesions in neonates with hypoxic-ischemic encephalopathy: magnetic resonance diagnosis and clinical outcome. World J Radiol.

[16] Corsiero E, Nerviani A, Bombardieri M, Pitzalis C (2016). Ectopic lymphoid structures: powerhouse of autoimmunity. Front Immunol.

[17] Zuo M, Wang AA, Gommerman JL (2025). Follicle on the roof: tertiary lymphoid structures in central nervous system autoimmunity. Immunol Rev.

[18] Christiansen MK, Kjær-Sørensen K, Clavsen NC (2023). Genetic analysis identifies the SLC4A3 anion exchanger as a major gene for short QT syndrome. Heart Rhythm.

